# Identification of p53-target genes in human papillomavirus-associated head and neck cancer by integrative bioinformatics analysis

**DOI:** 10.3389/fonc.2023.1128753

**Published:** 2023-04-04

**Authors:** Amal Bouzid, Muwaffaq Al Ani, David de la Fuente, Zainab Mohamed Al Shareef, Asif Quadri, Rifat Hamoudi, Natheer Al-Rawi

**Affiliations:** ^1^ Sharjah Institute for Medical Research, University of Sharjah, Sharjah, United Arab Emirates; ^2^ Ear Nose and Throat (ENT) Department, Tawam Hospital, Al-Ain, United Arab Emirates; ^3^ College of Medicine, University of Sharjah, Sharjah, United Arab Emirates; ^4^ Department of Anatomic Pathology, National Reference lab, Abu Dhabi, United Arab Emirates; ^5^ Division of Surgery and Interventional Science, University College London, London, United Kingdom; ^6^ Department of Oral and Craniofacial Health Sciences, College of Dental Medicine, University of Sharjah, Sharjah, United Arab Emirates

**Keywords:** head and neck cancer, oral squamous cell carcinoma, HPV, p53-target genes, MDM2, Prognosis biomarkers, bioinformatics

## Abstract

**Introduction:**

Head and neck cancer (HNC) is a highly prevalent and heterogeneous malignancy. Although extensive efforts have been made to advance its treatment, the prognosis remained poor with increased mortality. Human papillomaviruses (HPV) have been associated with high risk in HNC. TP53, a tumor suppressor, is the most frequently altered gene in HNC, therefore, investigating its target genes for the identification of novel biomarkers or therapeutic targets in HPV-related HNC progression is highly recommended.

**Methods:**

Transcriptomic profiles from three independent gene expression omnibus (GEO) datasets, including 44 HPV+ and 70 HPV- HNC patients, were subjected to integrative statistical and Bioinformatics analyses. For the top-selected marker, further in-silico validation in TCGA and GTEx databases and experimental validation in 65 (51 HPV- and 14 HPV+) subjects with histologically confirmed head and neck squamous cell carcinoma (HNSCC) have been performed.

**Results:**

A total of 498 differentially expressed genes (DEGs) were identified including 291 up-regulated genes and 207 down-regulated genes in HPV+ compared to HPV- HNSCC patients. Functional annotations and gene set enrichment analysis (GSEA) showed that the up-regulated genes were significantly involved in p53-related pathways. The integrative analysis between the Hub-genes identified in the complex protein-protein network and the top frequent genes resulting from GSEA showed an intriguing correlation with five biomarkers which are EZH2, MDM2, PCNA, STAT5A and TYMS. Importantly, the MDM2 gene showed the highest gene expression difference between HPV+ and HPV- HNSCC (Average log2FC = 1.89). Further in-silico validation in a large HNSCC cohort from TCGA and GTEx databases confirmed the over-expression of MDM2 in HPV+ compared to HPV- HNSCC patients (p = 2.39E-05). IHC scoring showed that MDM2 protein expression was significantly higher in HPV+ compared to HPV- HNSCC patients (p = 0.031).

**Discussion:**

Our findings showed evidence that over-expression of MDM2, proto-oncogene, may affect the occurrence and proliferation of HPV-associated HNSCC by disturbing the p53-target genes and consequently the p53-related pathways.

## Introduction

Over 800,000 people are diagnosed with head and neck cancer (HNC) every year. This makes HNC the sixth most frequent disease overall (1). Squamous cells that line the mucosal surfaces of the oral cavity, pharynx, and larynx are often the starting point for HNC. More than 90% of all malignancies of the head and neck are squamous cell carcinomas (HNSCC). The etiology of HNC is complicated and includes both genetic and environmental aspects (2), in addition to lifestyle variables, which differ between geographic locations (3). Alcohol intake and smoking are considered the two most classical risk factors for HNSCC (4). Moreover, in the past few decades, human papillomavirus (HPV) infection has been identified as a growing risk factor for HNSCC, defining a new class of tumors different from HPV-negative (HPV^-^) ones (5, 6). HPV-positive (HPV^+^) HNSCC is usually susceptible to radiation and anticancer medications and has a better prognosis, however, HPV^-^ HNSCC has genomic complexity and very common alterations in the tumor suppressor *TP53* and cell-cycle regulators (7).

The HPV family contains circular double-stranded DNA viruses of about 8000 base pairs encoding proteins responsible for viral replication (E1 and E2/E4), assembly (L1 and L2) and other accessory proteins (E5, E6 and E7). HPV16 and HPV18 contributed to around 85% of HPV^+^ HNC of worldwide cases whereas the remaining are caused by mainly HPV33, HPV35, HPV52, HPV45, HPV39, HPV58 (8, 9). The HPV infection can lead to a carcinogenic transformation of the infected mucosal epithelium (10) by escaping cell-cycle checkpoints through E6 and E7 mediated degradation of p53 and Rb proteins, respectively (11). Thus, the carcinogenic process in HPV malignancies is mostly associated with the E6 and E7 oncoproteins that together target various cellular pathways involved in the regulation of cell cycle control, apoptosis and cell polarity control networks (12). HPV-associated cancer has distinct profiles regarding gene/protein expression and genetic and epigenetic alterations. Multiple studies have demonstrated that the incidence and progression of HNSCC are closely associated with mutations and gene expression variations, including variation in *CTTN, D2HGDH, NLRP2, PEX11A, UPK* and *SERPINE1* and mutations in *CCR7, KL, LGR5* and *RORB*, which are associated with HNSCC prognosis (13, 14, 15). Moreover, numerous studies have confirmed the differential expression profiles between HPV-associated and non-HPV-associated HNSCC (16–18). However, very few diagnostic and prognostic markers have been reported with HPV-associated HNSCC subsets mainly limited to HPV, p16 and p53 (19), therefore, evaluating more biomarkers status, is highly required to predict the clinical outcome and survival of HPV-related HNSCC patients.

P53 protein, which is encoded by the *TP53* gene, is a transcription factor that regulates several cellular processes, including inflammation, cell senescence, cell metabolism, autophagy, and regulation of aberrant cell survival and death (20). Moreover, p53 is a tumor suppressor that plays a crucial function in the cellular response to stressors such as hypoxia and DNA damage and its inhibition outcomes in cells losing control of the mitotic cell cycle at the G2/M checkpoint (21). P53’s roles contributed to its interactions with other genes. Several earlier investigations found a broad network of P53 with several direct and indirect target genes (22). P53 protein is often unstable in the cell because it is continuously destroyed by MDM2, mouse double minute 2 homolog, also known as E3 ubiquitin-protein ligase Mdm2 (23). MDM2 cellular expression levels play a crucial function in the regulation of p53 in humans. Indeed, it has been confirmed that MDM2 amplification can result in p53 inactivation. (24), nevertheless, overexpression of MDM2 in tumors is frequently accompanied by poor prognosis (25). Thus, both MDM2 and HPV E6 oncoproteins play significant roles in regulating p53 in response to cellular stimuli, such as DNA damage and oncogenic signals (26).

The main objectives of this study are to use integrative bioinformatics and network interaction-based methodologies to compare the whole transcriptome of HPV^+^ and HPV^-^ HNSCC patients and identify the possible molecular interactions and hub genes that may be associated with HPV-related HNSCC grading and prognosis.

## Patients and methods

### Transcriptomic analysis data source

The gene expression microarray datasets GSE3292, GSE6791 and GSE55542 were retrieved from the Gene Expression Omnibus (GEO) database. Only gene expression datasets that had been performed in HNC patients’ tumor samples and involved the HPV status were selected. In total 114 samples were analyzed, including 44 HPV^+^ and 70 HPV^-^ HNC patients (**Supplementary Table 1**).

### Data processing of DEGs between HPV^+^ and HPV^-^ HNC patients

Quality control analysis, technical heterogeneity and batch effect exclusion, data background correction and data normalization are performed for all HNC samples. To detect the differentially expressed genes (DEGs) between HPV^+^ and HPV^-^ HNC patients, the *LIMMA* package of R was applied (27). Genes that met the cutoff criteria with fold change (FC) FC ≥ 2 and p-value (*p) < 0.05* were considered as significant DEGs between HPV^+^ and HPV^-^ HNC patients. Overall patient’s transcriptome data, for a given gene, and the maximum expression value of the multiple probes were considered. The processed expression datasets were merged into a common expression matrix based on the gene names.

### Functional enrichment analysis of the DEGs between HPV^+^ and HPV^-^ HNC patients

We performed Gene Ontology (GO) and Kyoto Encyclopedia of Genes and Genomes (KEGG) pathway enrichment analyses using hypergeometric tests to uncover the putative biological roles of the DEGs between HPV^+^ and HPV^-^ HNC patients. Genes were annotated based on their associated Biological Processes (BP), Molecular Function (MF), and Cellular Component (CC) gene ontologies.

### Gene set enrichment analysis

The Gene Set Enrichment Analysis (GSEA) was performed to identify gene sets that were differentially regulated between HPV^+^ and HPV^-^ HNC patients. Normal and absolute gene set enrichment analyses were conducted on the DEGs as stated before (28). The analyzed gene sets included the collections C2 (curated), C4 (computational), C5 (gene ontology), C6 (oncogenic signatures), C7 (immunologic signatures) and C8 (cell type signature) which are obtained on the latest updated version v2022 from the Human Molecular Signatures Database (MSigDB) (https://www.gsea-msigdb.org). In addition to the subset of p53 target genes which was collected from online pathway databases, publications and through bioinformatics search (**Supplementary Table 2**). The significant GSEA results were selected according to the nominal *p* < 0.05. Additionally, the leading-edge analysis was carried out to detect the biologically potential gene subset among the gene sets that are differentially regulated between HPV^+^ and HPV^-^ HNC patients.

### Construction of protein-protein interaction network

To assess the potential Protein-Protein Interaction (PPI) network of the DEGs between HPV^+^ and HPV^-^ HNC patients, the STRING database (v11.5) was used (https://string-db.org/). The DEGs were mapped, and the corresponding PPI pairs were identified with the highest confidence interaction score of 0.9. Next, to identify the target genes, the PPI network was visualized using Cytoscape software (https://cytoscape.org/). In the network, nodes showing a higher number of links are apt to preserve the stability of the whole network based on the edge-connectivity concept. The cytoHubba application (v0.1) was used to find the top hub nodes ordered by degree of interactions using the Degree method.

### Patients’ samples collection

To validate the protein expression of the selected potential genes from the *in-silico* transcriptomic analysis, a total of 65 patients with oral squamous cell carcinomas (OSCC) were collected, including 14 HPV^+^ and 51 HPV^-^ OSCC samples. The case classification of HPV status was based on the immunostaining of the p16 protein which is considered as a surrogate marker to diagnose high-risk HPV infection in OSCC according to the College of American Pathologists (29). All patients were selected and retrieved after ethical review and approval from the Tawam Hospital (Al-Ain, UAE) ethical committee (REC: AA/AJ/556). Detailed clinicopathological information was assembled from the patient’s medical records at Tawam Hospital. Two expert histopathologists (NA-R and AQ) initially examined the H&E-stained tissue sections to confirm the histological diagnosis. The tumors are originated from the tongue, floor of the mouth, cheek, gingiva, palate, or retromolar regions. Since the vermilion borders of the lip and the pharyngeal complex are not considered parts of the oral cavity, they were excluded.

### Clinical and histopathological evaluation

The clinical and histopathological evaluation of the OSCC samples was performed based on the American Joint of cancer Committee on Cancer Staging (AJCC) classification (Sixth edition). All clinical information regarding the pathological tumor node metastasis (pTNM) staging of the original tumor, the level of cancer invasion and metastasis, the resection margin of cancer, the perineural invasion (PNI) and the lymphovascular invasion (LVI), were obtained from the patient’s histopathology reports.

### Tissue microarrays

Each tumor’s invasive front was identified, and a donor paraffin block’s core (0.5 cm) was considered for the experiment. From the 65 OSCC samples, 16-17 cores per block of tissue microarrays (TMA) were placed on four paraffin blocks. Initial H&E slides that subsequently revealed a limited tumor area were deleted from the following procedure analysis.

### Immunohistochemistry

Paraffin sections were cut at 4 um thickness, placed on positively charged slides, and dried in an oven for 30 minutes at 70°C. Deparaffinization, rehydration, and target retrieval were performed in the PT Link (Dako) using a 3-in-1 procedure. Details of the immunohistochemistry (IHC) staining protocol for the used antibodies for p16, MDM2 and p53 were described in **Supplementary Table 3**. IHC was performed on a Benchmark-ULTRA fully automated staining instrument (Roche Diagnostics, USA) using an Ultra View Universal DAB Detection kit from (Ventana). The antibodies were revealed by DAB and then counterstained with Meyer’s Hematoxylin and bluing reagent. Image acquisition and analysis were performed as previously described (30). Validation of Image J analysis for the three above-mentioned markers was performed by two observers who are experienced pathologists (NA-R and AQ). Controlling of threshold level was scaled and maintained without much adjustment (31). HPV positivity was considered when >40% of malignant epithelial cells were positive for P16 (32). The necrotic area of the specimen was not taken into account as previously described (31, 33).

### Bioinformatics analysis

The potential role of MDM2 in HPV-associated HNSC was further evaluated based on the Cancer Genome Atlas (TCGA) and the Genotype-Tissue Expression (GTEx) data resources using the Gene Expression Profiling and Interactive Analysis database (GEPIA) (http://gepia.cancer-pku.cn/), which represents the gene expression alterations in large cohort cancer samples, and the Tumor IMmune Estimation Resource (TIMER) (https://cistrome.shinyapps.io/timer/), which presents a comprehensive analysis of tumor-infiltrating immune cells and UALCAN resource (http://ualcan.path.uab.edu/index.html), which gives access to different publicly available cancer OMICS data. The survival analysis of the *MDM2* gene was evaluated in HNSCC patients with high mutation burden, using the Kaplan-Meier plots.

### Statistical analysis

The statistical analysis was performed using GraphPad prism software v5.0.1 (GraphPad Software, La Jolla California USA). Statistical significance between the protein expression and clinical parameters was analyzed using the t-test. Pearson’s correlation was applied to evaluate the possible correlation between protein expression and different clinical parameters across HPV^+^ and HPV^-^ OSCC patients. A two-way *p* < 0.05 was considered statistically significant.

## Results

### Transcriptional profiling distinguishes between HPV^+^ vs HPV^-^ HNSCC patients

**Figure 1:**
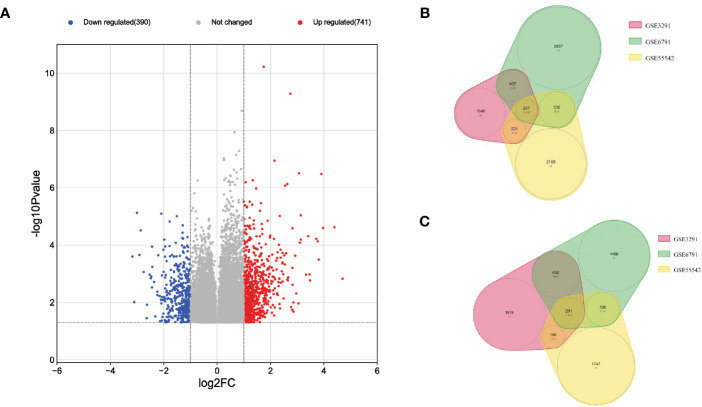
Visualization of the DEGs across the three transcriptomic datasets of HNSCC patients. **(A)** Volcano plot with the blue points representing 207 down-regulated genes and the red points representing 291 up-regulated genes in HPV^+^ compared to HPV^-^ HNSCC patients, Venn Diagrams of; **(B)** down-regulated and **(C)** up-regulated genes in HPV^+^ compared to HPV^-^ HNSCC patients.

After processing the whole transcriptomic data from the three preselected datasets GSE3291, GSE6791 and GSE55542, the merged data showed a total of 498 common differentially expressed genes (DEGs) (FC ≥ 2, *p* < 0.05) between the HPV^+^ compared to HPV^-^ HNSCC patients across the different datasets (**Figure 1A**). The DEGs included 207 down-regulated genes (**Figure 1B**) and 291 up-regulated genes (**Figure 1C**) in HPV^+^ compared to HPV^-^ HNSCC patients.

### Aberrant signaling and transcriptional regulation pathways in HPV-associated HNSCC

**Figure 2:**
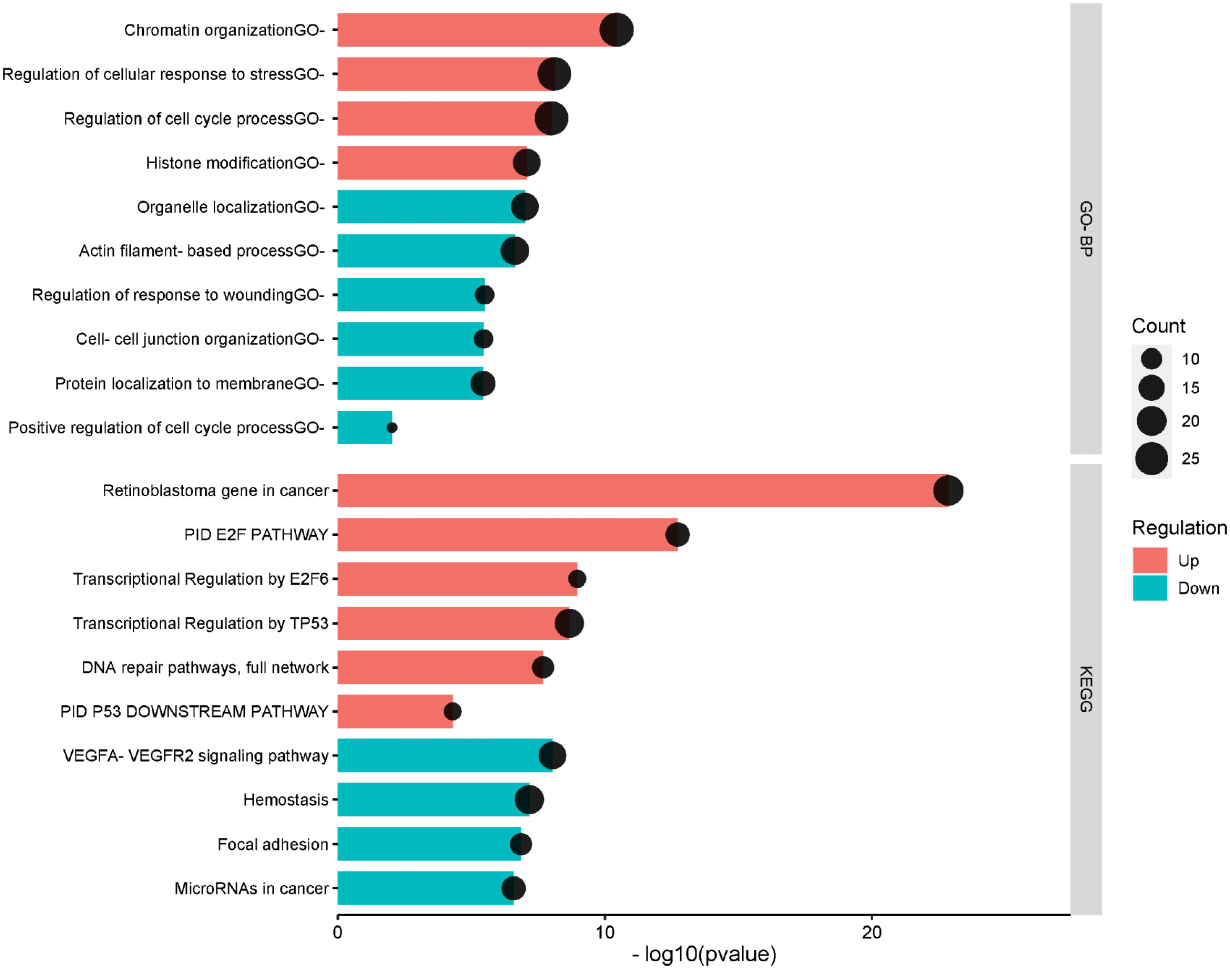
The top significantly enriched Gene ontology biological process and KEGG pathways of the differentially expressed genes in HPV+ compared to HPV- HNSCC patients. The pathways are presented in the red shade for the upregulated and the blue shade for the down-regulated. The vertical axis is the GO-BP: GO Biological Processes or KEGG: KEGG Pathway term. The horizontal axis is the transformed p-value (-log10pvalue). The count presents the genes count in the corresponding terms.

To assess the biological functions related to the identified DEGs, functional annotation and pathway analysis have been performed for GO and KEGG analyses. The up and down-regulated genes were separately annotated. The top 20 enriched terms including the related up/down-regulated genes in HPV^+^ compared to HPV^-^ HNSCC are presented in **Figure 2**. The top 20 enriched terms and the related genes is provided in **Supplementary Table 4**. The most up-regulated genes in HPV^+^ compared to HPV^-^ HNSCC were significantly enriched in the cell cycle regulation process including the PID E2F pathway (*p*= 1.89E-13), Transcriptional Regulation by E2F6 (*p*= 1.09E-09) and Regulation of cell cycle process (*p*= 9.99E-09), DNA replication and repair including DNA repair pathways (*p*= 2.06E-08), Chromatin organization (*p*= 3.6E-11) and Histone modification (*p*= 8.36E-08) and in the p53 tumor suppressor protein regulation (*p*= 5.02 E-05) including the Transcriptional Regulation by TP53 (*p*= 2.15E-09), PID P53 Downstream pathway and Regulation of cellular response to stress (*p*= 2.15E-09). While the down-regulated genes were mainly annotated in the VEGFA-VEGFR2 signaling pathway (*p*= 9.18E-09), Hemostasis (*p*= 6.71E-08) and Focal adhesion (*p*= 1.38E-07).

### Identification of protein-protein interaction network, clusters and hub genes of the DEGs in HPV^+^ vs HPV^-^ HNSCC patients

**Figure 3:**
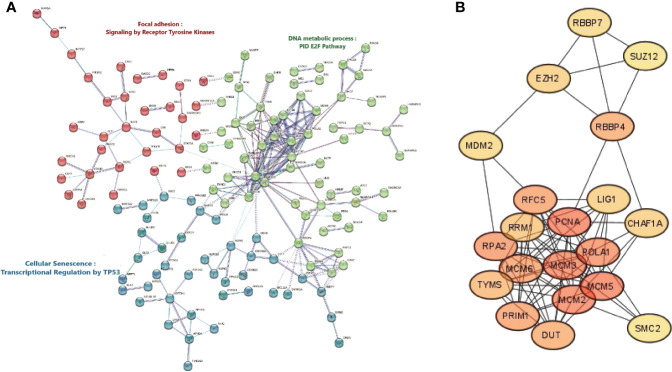
Protein-Protein Interactions network, Clustering analysis, and Hub Genes identification. **(A)** Cluster analysis of the protein-protein interaction network complex of the differentially expressed genes in HPV^+^ compared to HPV^-^ HNSCC patients using the STRING database. The network is portioned into 3 clusters based on the k-mean clustering algorithm. The solid and the dotted lines indicate a connection inside the same and other clusters, respectively. Different colors of edges indicate the diverse type of protein-protein interaction including Cyan: curated related databases, Pink: experimentally determined, Blue: gene co-occurrence, Black: co-expression, and Light blue: protein homology. **(B)** Discovering top scoring twenty Hub genes in HNSCC, selected by a topological analysis with Degree method by the CytoHubba in Cytoscape.

To identify the protein connections amongst the differentially expressed genes between HPV^+^ vs HPV^-^ HNSCC patients, the PPI-network interactions were constructed at the highest confidence of 0.9, defined as the minimum required interaction score (**Figure 3A**). Overall, the protein-protein interaction network has a p-value less than 1.0E-16, a total of 476 nodes with an average node degree of 0.929. The interaction enrichment showed a total of 221 edges while only 106 were expected, which indicates that the evaluated proteins are biologically interconnected as a group. For details of PPI networks and to identify the top protein complexes, we assumed that proteins under the same cluster probably share similar biological functions. Thus, the total proteins were clustered into the three highest-scored groups based on the clustering coefficient, using k-means clustering. **Figure 3A** showed that; Cluster 1 (colored in red), with PPI enrichment *p*-value = 1.13E-07, consisting of 27 nodes was highly enriched in focal adhesion and signaling by receptor tyrosine kinases. Cluster 2 (colored in green), with PPI enrichment *p*-value < 1.0E-16, consisting of 58 nodes was mainly associated with the DNA metabolic process and E2F regulation pathway. Cluster 3 (colored in blue), with PPI enrichment *p*-value = 2.97E-12, consisting of 38 nodes was related to cellular senescence and transcription regulation by TP53. The proteins and their node degree related to each cluster are listed in **Supplementary Table 5**. Moreover, the PPI network was further explored to identify the hub genes, showing the highest number of connections in the network, using the Degree method under the CytoHubba plugin. The top 20 hub genes with high scoring were identified based on the degree of each protein node, including *PCNA, MCM5, MCM3, MCM2, POLA1, RPA2, MCM6, PRIM1, RFC5, RBBP4, DUT, TYMS, RRM1, EZH2, LIG1, CHAF1A, RBBP7, MDM2, SUZ12*, and *SMC2* (**Supplementary Table 6**). The sub-network interaction between the top 20 hub genes is presented in **Figure 3B**.

### Differential expression of p53-target genes in HPV-related HNSCC

**Figure 4:**
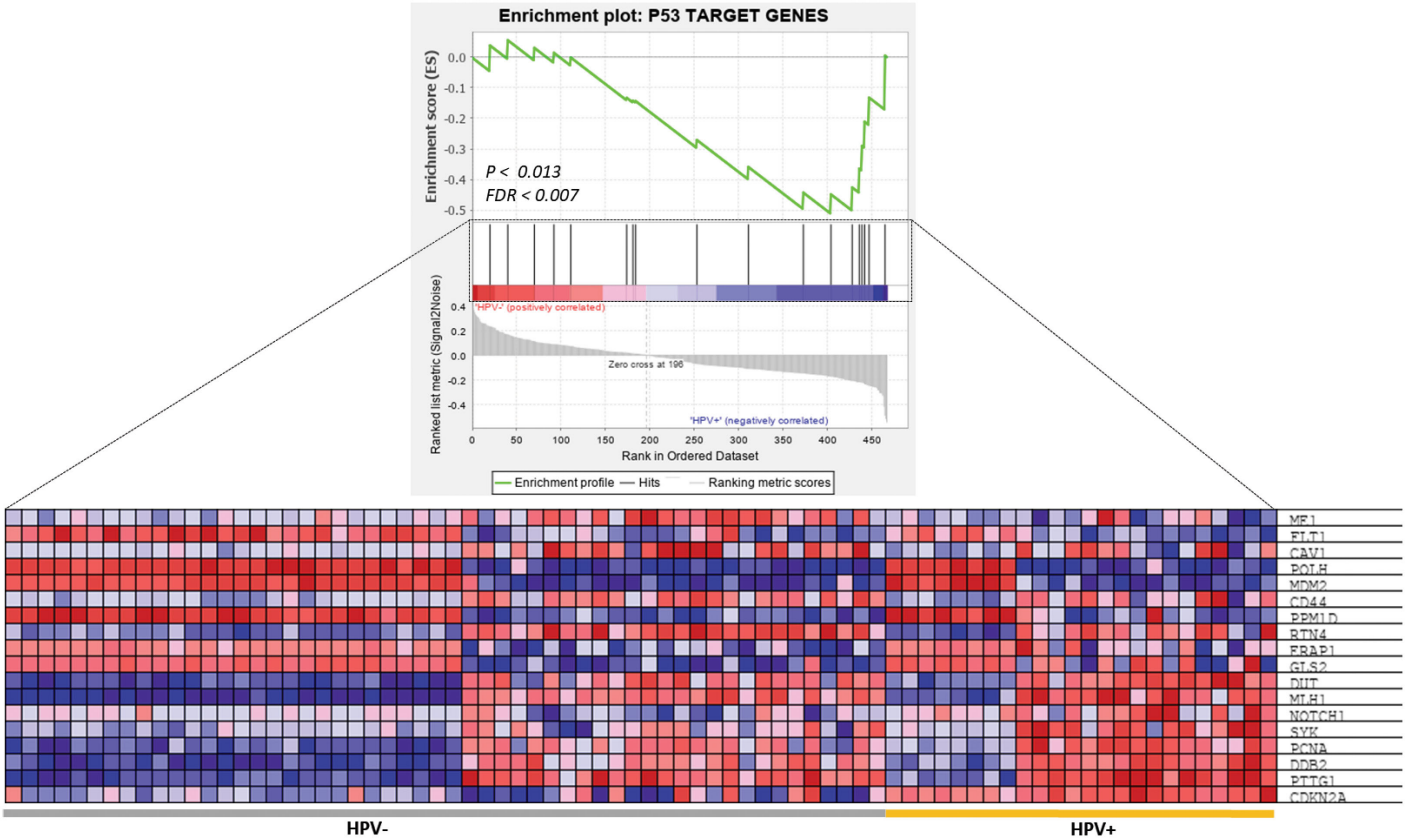
GSEA of P53 target genes in HPV^+^ and HPV^-^ HNSCC patients. The top image shows the distribution of P53 target genes based on the rank position after enrichment analysis. The bottom image is a heatmap presentation showing the expression profiling of 18 enriched P53 target genes between HPV^+^ and HPV^-^ HNSCC samples.

As anticipated, the GSEA showed that a subset of the p53-target genes was highly significantly enriched (ES = 0.558, *p* = 0.013 and FDR = 0.007) in the patients with HPV-related HNSCC (**Figure 4**). The leading-edge analysis revealed that 18 core genes were significantly enriched in HPV-related HNSCC patients, including *CDKN2A, DDB2, DUT, ERAP1, GLS2, MDM2, MLH1, NOTCH1, PCNA, POLH, PPM1D, PTTG1, SYK* that were up-regulated in HPV^+^ HNSCC patients, however, *CAV1, CD44, FLT1, ME1*, and *RTN4* were up-regulated in HPV^-^ HNSCC patients (**Figure 4**).

### p53-target genes potentially derive the differential signature of significant gene sets between HPV^+^ and HPV^-^ HNSCC patients

To gain a better understanding of the potential distinctness in molecular signatures and biological mechanisms between HPV^+^ and HPV^-^ HNSCC. The total DEGs between HPV^+^ and HPV^-^ HNSCC patients were subjected to GSEA. First, the absolute GSEA was performed on 29,122 gene sets encompassing complex molecular mechanisms, different cellular pathways, biological processes and molecular functions. After excluding those with general terms or those including less than twenty genes, a total of 40 potent significant gene sets were differentially enriched between HPV^+^ and HPV^-^ HNSCC patients (*p* < 0.05). The details of these significant gene sets are listed in **Supplementary Table 7**. Overall, in the identified significant gene sets, a broad overlap was noticed among the different gene sets with HPV-associated HNSCC-related cellular pathways or molecular mechanisms. Therefore, we grouped them mainly based on their involvement in the p53 pathway, Epithelial-Mesenchymal Transition, proliferation, GPR signaling and MAPK pathway, immune response/inflammation and metabolic process. Additionally, leading-edge analysis was carried out to detect the core-subset genes that potentially underlie the significant enrichment of the corresponding gene set and play substantial biological roles. Next, the gene frequency analysis was computed based on the occurrence of a gene across all the enriched leading-edge core genes from the 40 significantly over-represented gene sets. Based on the 50-percentile cutoff of the frequency values, 51 top frequent genes were identified (**Table 1**). Interestingly, the p53-target genes were frequently listed in the core subset genes across several activated pathways, and it is noteworthy that the top two frequent genes across all significant gene sets are *NOTCH1* (24 occurrences) and *CAV1* (23 occurrences), which reinforces the previous analysis showing the important subset of p53-target genes in HPV-associated HNSCC.

**Table 1:** The Top Frequent genes based on the count in the activated pathways using GSEA in HPV^+^ compared to HPV^-^ HNSCC.

Num	Gene name	Count	Num	Gene name	Count	Num	Gene name	Count
1	*NOTCH1*	24	19	*HMGA2*	12	37	*POU4F1*	8
2	*CAV1*	23	20	*RIPK2*	12	38	*PTTG1*	8
3	*EZH2*	20	21	*STAT5A*	12	39	*RBBP7*	8
4	*PDGFB*	18	22	*SYK*	12	40	*SNX6*	8
5	*SMARCA4*	18	23	*TYMS*	12	41	*BCL11A*	7
6	*THBS1*	17	24	*NCK1*	11	42	*BRD8*	7
7	*CAV2*	16	25	*PHIP*	11	43	*CBS*	7
8	*EDNRA*	15	26	*SP1*	11	44	*FERMT2*	7
9	*PCNA*	15	27	*TIMELESS*	11	45	*HELLS*	7
10	*IRS1*	14	28	*TSC1*	11	46	*IL31RA*	7
11	*PRKCE*	14	29	*CDC42*	10	47	*KLF7*	7
12	*ROCK1*	14	30	*NAMPT*	10	48	*LMO4*	7
13	*SHC1*	14	31	*DHFR*	9	49	*PAPPA*	7
14	*AKR1C1*	13	32	*FLT1*	9	50	*RTN4*	7
15	*BCL2L11*	13	33	*MDM2*	9	51	*SRSF6*	7
16	*CDKN2A*	13	34	*CD44*	8			
17	*HMGB2*	13	35	*GKAP1*	8			
18	*PIK3R3*	13	36	*MYO5A*	8			

### Integrative analysis showing potential biomarkers and pathways behind the progression of HPV-associated HNSCC

**Figure 5:**
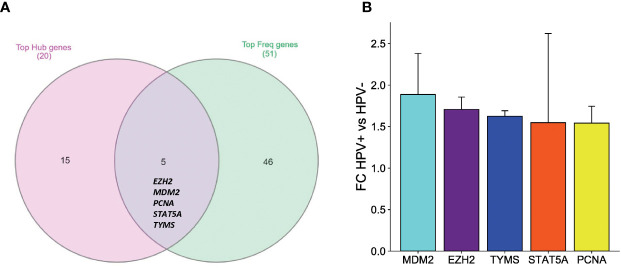
An integrative analysis of the key DEGs in HPV^+^ compared to HPV^-^ HNSCC patients. **(A)** Venn diagram showing the overlapping between the top hub genes generated by PPI analysis and the top frequent genes resulting from the GSEA analysis. **(B)** Bar graph comparing the gene expression fold changes identified by the integrated network analysis. Five upregulated genes in HPV^+^ compared to HPV^-^ HNSCC patients. The standard deviation error bar for each is presented showing the variability of each gene expression amongst the three datasets.

To identify key biomarker genes and pathways involved in the progression of HPV-associated HNSCC, integrative analysis on the transcriptomics analyses was performed with several bioinformatics analyses. First, the comprehensive and integrated analysis was performed on the top significant pathways which resulted from the functional annotation of the DEGs, clustering of the top protein complexes and GSEA to show that the most marked observation to emerge from the data comparison was p53-related pathways and regulation. The second integrated analysis was carried out on the significant resulted genes from the top 20 hub genes analysis and the top frequent genes in GSEA to show intriguing correlation with five biomarkers which are *EZH2, MDM2, PCNA, STAT5A* and *TYMS* (**Figure 5A**). To further assess the expression stability of these genes across the different datasets, knowing that their ethnicities are different American, European-American and African American, the gene expression fold changes between HPV^+^ and HPV^-^ HNSCC were evaluated. Importantly, the *MDM2* gene showed the highest gene expression difference between HPV^+^ and HPV^-^ HNSCC (Average log2FC = 1.89, Average of absolute deviation = 0.49) (**Figure 5B**). These results have further strengthened our assumption that over-expression of *MDM2*, proto-oncogene, may affect the occurrence and proliferation of HPV-associated HNSCC by disturbing the p53 target genes and consequently the p53-related pathways.

### *In-silico* validation of *MDM2* involvement in HPV-associated HNSCC

In order to provide additional evidence for our hypothesis, exhaustive *in-silico* validation of the *MDM2* involvement in HPV-associated HNSCC was performed. First, the mRNA level of the *MDM2* gene was assessed in a large sample size of HNSCC tumor samples (519 samples) compared to normal tissues (44 samples) using data from TCGA and GTEx in GEPIA. Importantly, the mRNA levels of *MDM2* expression were significantly higher in HNSCC tissues than in normal tissues (*p* < 0.05) (**Supplementary Figure 1A**). In addition, the *MDM2* expression was evaluated in HNSCC based on the HPV status using data from TCGA, including 41 HPV^+^ HNSC, 80 HPV^-^ HNSCC patients and 44 normal cases. As expected, the *MDM2* showed a very significant over-expression in HPV^+^ than in HPV^-^ HNSCC patients (*p* = 2.39E-05) and in normal samples (*p* = 2.81E-07), however, only a slight difference was observed between HPV^-^ HNSCC patients and normal samples (*p* = 0.03) (**Supplementary Figure 1B**). These results were consistent with our findings from GEO datasets and further make evidence that the *MDM2* gene is a key biomarker to distinguish between HPV^+^ and HPV^-^ HNSCC patients. Moreover, to assess the prognostic potential of *MDM2*, the overall survival analysis was performed in a cohort of HNSCC patients and in a cohort of HNSCC based on the HPV status using the Kaplan-Meier plots. The results reveal that a high level of *MDM2* (HR= 1.44, 95%CI (1-2.06), *p* = 0.046) is correlated with poor overall survival in HNSCC patients (**Supplementary Figure 2A**). However, the high level of *MDM2* demonstrates significantly increased overall survival (HR= 0.461, 95%CI (0.291-0.728), *p =* 0.002) in HNSCC HPV^+^ patients, while no significant association (*p* = 0.263) was reported between the *MDM2* expression and the overall survival of the HNSCC HPV^-^ patients (**Supplementary Figure 2B**).

### HNSCC patients’ characteristics

The clinical and histopathological evaluation of the collected OSCC samples was performed based on the AJCC classification (Sixth edition). **Table 2** summarizes the characteristics of the 65 OSCC patients who were recruited in this study. The cohort included 70.8% males and 29.2% females. The patients ranged in age from 18 to 78 years old, with a median age of 57 and showing that about 61.5% of the patients were under the age of 60. Cigarette smoking habits constitute 21.5% of the patients while smokeless tobacco habits concern only 6% of the patients. The most common site of OSCC was the tongue (63.1%), followed by the cheek (13.8%) and Jawbones (9.2%). A total of 13.8% of the cohort had cancer in more than one place while about 64.6% of the cases were at the late stage (TNM stages III and IV). Recurrent tumors were observed in 21.5% of cases only. Lymph node metastasis was found in 35.4% of patients with single or multiple ipsilateral spread. About 50% of cases were well differentiated SCC with PNI in 28.6% of cases.

**Table 2:** Demographic and Clinical characteristics of the OSCC patients.

	Total sample		HPV^+^ patients		HPV^-^ patients		
	N	%	N	%		N	%
Gender							
**Female**	19	29.2	2	14.3		17	33.3
**Male**	46	70.8	12	85.7		34	66.7
**Total**	65	100.0	14	100		51	100
Age group (years)							
**< 60**	40	61.5	3	21.4		37	72.5
**60+**	25	38.5	11	79.6		14	27.5
**Total**	65	100.0	14	100		51	100
Tobacco use							
**None**	47	72.3	9	64.2		38	74.5
**Cigarette smoking**	14	21.5	5	35.8		9	17.6
**Smokeless tobacco use**	4	6.2	0	0		4	7.8
**Total**	65	100.0	14	100		51	100
Primary or recurrent cancer site							
**Tongue**	41	63.1	7	50		34	66.7
**cheek**	9	13.8	0	0		9	17.6
**Jaw bones**	6	9.2	4	28.6		2	3.9
**Multiple**	9	13.8	3	21.4		6	11.8
**Total**	65	100.0	14	100		51	100
Multiple Vs Single tumor							
**Single**	56	86.2.	11	78.5		42	82.3
**Multiple**	9	13.8	3	21.5		9	17.7
**Total**	65	100.0	14	100		51	100
T stage (tumor size)							
**T1**	22	33.8	4	28.6		18	35.3
**T2**	15	23.1	3	21.4		12	23.5
**T3**	8	12.3	2	14.3		6	11.7
**T4**	20	30.8	5	35.7		15	29.5
**Total**	65	100.0	14	100		51	100
N Staging (Cervical LN metastasis)							
**No**	42	64.6	8	57.1		34	66.7
**N1**	6	9.2	2	14.3		4	7.8
**N2**	13	20.0	2	14.3		11	21.6
**N3**	4	6.2	2	14.3		2	3.9
**Total**	65	100.0	14	100		51	100
Cervical LN metastasis (N1-3)							
**Negative**	42	64.6	9	64.3		33	
**Positive**	23	35.4	5	35.7		18	
**Total**	65	100.0	14	100		51	
Distant metastasis to the lung or liver							
**Negative**	32	88.9	14	100		18	81.8
**Positive**	4	11.1	0	0		4	18.2
**Total**	36	100.0	14	100		22	100
TNM stage							
**Stage I**	15	23.1	4	28.6		11	21.5
**Stage II**	8	12.3	3	21.4		5	9.8
**Stage III**	12	18.5	2	14.3		10	19.6
**Stage IV**	30	46.2	5	35.7		25	49
**Total**	65	100.0	14	100		51	100
Late stage cancer (Stage 3-4)							
**Early stage (1-2)**	23	35.4	7	50		16	31.3
**Late stage (3-4)**	42	64.6	7	50		35	68.7
**Total**	65	100.0	14	100		51	100
Hisological tumor grade							
**G1: Well differentiated**	33	50.8	7	50		26	50.9
**G2:moderately differentiated**	29	44.6	6	42.8		23	45.1
**G3: poorly differentiated**	3	4.6	1	7.2		2	4
**Total**	65	100.0	14	100		51	100
Lymphovascular invasion under microscopic examination of tumor whether present or not							
**Negative**	46	82.1	12	14.3		34	80.9
**Positive**	10	17.9	2	85.7		8	19.1
**Total**	56	100.0	14	100		42	100
Perineural invasion by tumor cells under histological examination							
**Negative**	40	71.4	11	78.5		29	69.1
**Positive**	16	28.6	3	21.5		13	30.9
**Total**	56	100.0	14	100		42	100

### Validation of MDM2 protein expression

**Figure 6:**
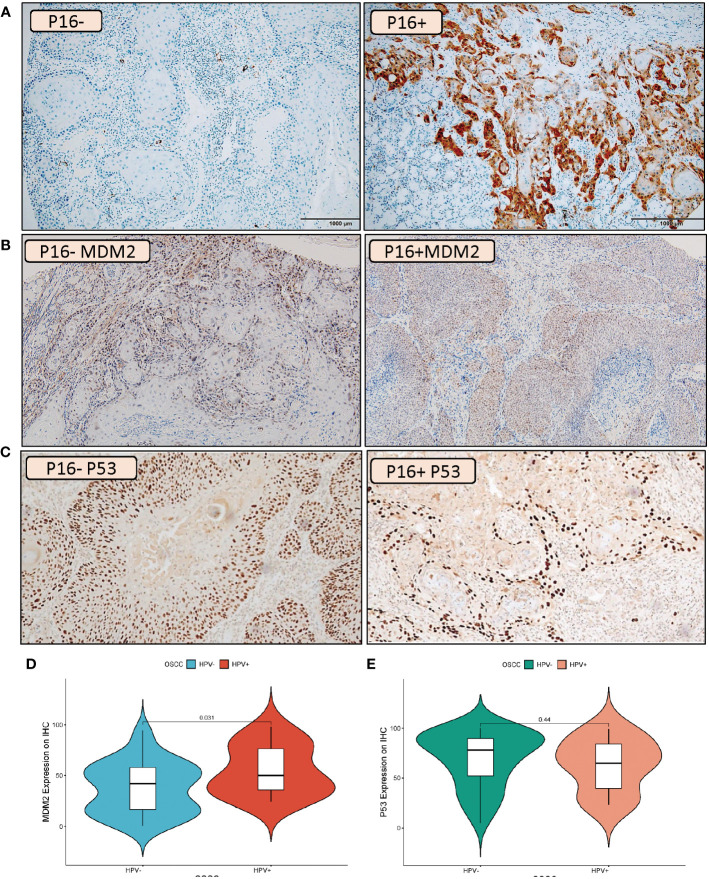
IHC Validation of the MDM2 and P53 expression levels in HPV^+^ and HPV^-^ OSCC patients. Example of IHC analysis (10X) of P16 **(A)**, MDM2 **(B)** and P53 **(C)** in oral squamous cell carcinoma patients. For MDM2, the left side of the image represents HPV^-^ tumors which illustrate a low degree of IHC staining while the right side of the image represents HPV^+^ tumors which indicates much more IHC staining. For P53 no distinct variation was observed between IHC staining in HPV- tumors (left side) and HPV^+^ tumors (right side). Violin plot of MDM2 **(D)** and P53 **(E)** protein expression changes in HPV^+^ compared to HPV^-^ HNSCC patients. The statistically significant expression differences between HPV^+^ and HPV^-^ OSCC samples were evaluated using a t-test (*p* < 0.05).

A tissue microarray was used for assessing the protein levels of MDM2 in HPV^+^ and HPV^-^ OSCC patients (**Figure 6A**). The IHC scoring showed a low level of IHC staining in HPV^-^ OSCC patients with an average expression of 37.87%, while, the HPV^+^ OSCC disclosed a high level of MDM2 expression with much more IHC staining indicating an average expression of 55.04% (**Figure 6B**). Importantly, according to the tissue microarray analysis, the MDM2 protein expression was significantly higher in HPV^+^ compared to HPV^-^ HNSCC patients (*p = 0.031)*, (**Figure 6D**). Additionally, considering the close relationship between MDM2 and p53, we also evaluated the protein expression of p53 in HPV^+^ and HPV^-^ OSCC patients (**Figure 6C**). Notably, no significant variation (*p* = 0.44) has resulted between HPV^+^ and HPV^-^ OSCC patients (**Figure 6E**).

### Correlation of MDM2 expression with clinicopathological parameters in HPV-associated HNSCC patients

**Figure 7:**
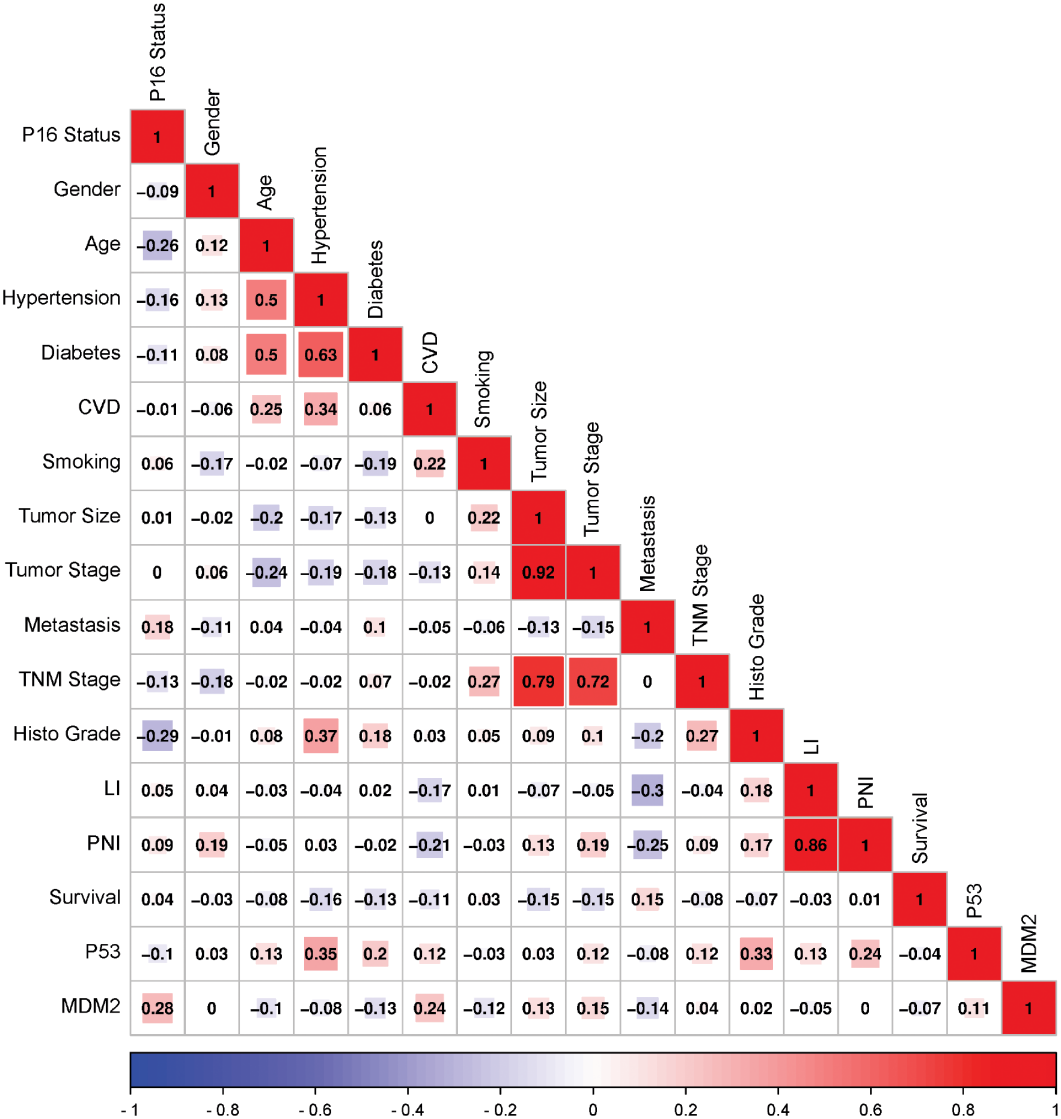
Correlation matrix among MDM2 and P53 expressions and various demographic and Clinical characteristics of the HNSCC patients. Analysis was performed by Pearson’s Correlation, *p* ≤ 0.05. Red indicates a positive correlation, and blue indicates a negative correlation. Darker colors are related to stronger correlation coefficients. CVD, Cardiovascular diseases; LI, Lymphovascular invasion; PNI, Perineural invasion.

Mutual correlations were identified between MDM2 protein expression and different demographic clinical characteristics of the HNSCC patients, using Pearson’s correlation (p ≤ 0.05). As shown in **Figure 7**, positive correlations have resulted importantly between MDM2 and HPV status, cardiovascular diseases, tumor size and tumor stage. Also, a slight positive correlation was reported between p53 and MDM2 expressions. While negative correlations have resulted between MDM2 and diabetes, smoking, metastasis and survival.

## Discussion

Lack of knowledge about key genes and molecular pathways that drive the onset and progression of HPV-associated HNSCC has prevented early identification and therapy despite the growing body of research on the disease. Therefore, using bioinformatics analysis helps to get an in-depth understanding of the molecular keystones of HPV-associated HNSCC progression which are extremely needed for prediction, early detection, and therapy. To date, computational and high-throughput approaches have become more powerful and relevant to identify biomarkers in cancer study that helps in the understanding of carcinogenesis by integrating omics data (34). Among several bioinformatics approaches, we applied the absolute GSEA which is a useful tool for deducing biologically relevant information from large transcriptomics data, and is most robust as it targets a group of genes that share a category of functions or biological pathways, rather than individual genes. Particularly, the absolute GSEA has been widely applied in different types of cancers to identify potential tumor biomarkers involved in tumorigenesis, diagnosis and therapeutic (28, 35, 36).

In this study, systematic bioinformatics analyses were used to compare and integrate transcriptomics data between HPV^+^ and HPV^-^ -HNSCC. Importantly, we showed that p53-target genes, among others *MDM2* was a central biomarker, potentially deriving the differential signature of significant molecular pathways between HPV^+^ and HPV^-^ HNSCC patients which is consistent with previous findings showing that *MDM2* genetic aberrations and related pathways play a central role in the carcinogenesis of head and neck cancers in addition to a particular correlation with HPV status (37). Furthermore, *MDM2* has been identified as a crucial biomarker in many malignancies e.g lung cancer, breast cancer, liver cancer, esophagogastric cancer and colorectal cancer as it plays an important role in the control of p53 and the onset of cancer (38). Hence, our findings further support the idea that *MDM2* may serve as a prognostic indicator for HNSCC in general and HPV^+^ HNSCC in particular.

From our findings, the transcriptional profiling between HPV^+^ vs HPV^-^ HNSCC patients revealed that among the five identified biomarkers, *MDM2* showed the highest gene expression (Average log2FC = 1.89) difference between HPV^+^ and HPV^-^ HNSCC. *MDM2*, a proto-oncogene, is amplified in 25–40% of human malignancies. The reported frequency of *MDM2* overexpression is high in HNSCC, ranging from 40 to 80%. *MDM2* can be overexpressed *via* either gene amplification, improved transcription, or post-transcriptional mechanisms (39, 40). Unsuitable excesses of *MDM2* could lead to exaggerated silencing of p53, revoking its shielding tumor suppressor effects. Despite that several other proteins are involved in the control of p53 stability in HNSCC, the p53-MDM2 is considered an important integrated component of a complex cellular network of the p53-targets genes (41). The p53-MDM2 paradigm has been well evaluated to highlight the association between a tumor suppressor gene functions as a transcription factor and an oncogene functioning as an E3 protein ligase but the outcome of MDM2 overexpression changes in the p53 downstream genes has still poorly understood.

The immunohistochemistry validation in HPV^+^ and HPV^-^ OSCC patients showed that MDM2 was positive in 41.57 % of the cells, indicating that more than half of the tumors lack or had low levels of MDM2 protein. In contrast, the MDM2 protein was consistently detected in basal and parabasal cells of morphologically normal epithelium outside of the invasively developing tumor. The mean value of MDM2 expression was substantially greater in the HPV^+^ group than in the HPV^-^ group (60.29 ± 25.06 vs. 36.89 ± 23.40). These findings provide considerable insight into the prognostic role of MDM2 in HPV^+^ HNSCC patients.

**Figure 8:**
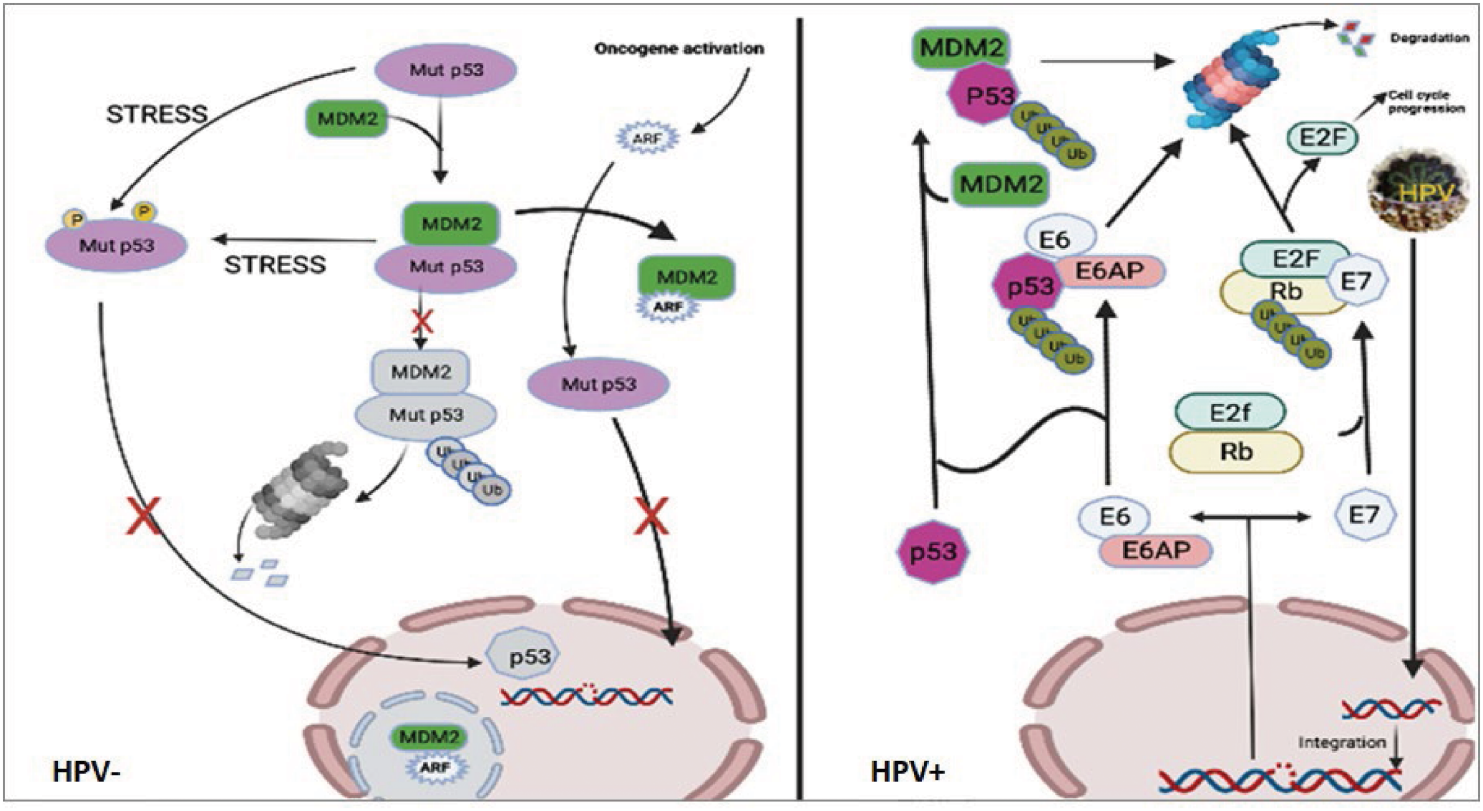
Pathways of p53 signaling in cells with HPV- or HPV^+^ cells. The left figure showed that although mutant p53 follows the same routes as wild-type p53, in HPV- cells the mutation in p53 prevents transcription activation from occurring downstream. The right figure showed that upon infection with HPV, the viral DNA integrates into the host’s genome, where it is transcribed to produce new viral particles. Additionally, viral E6 and E7 enzymes are translated, resulting in the degradation of p53 and Rb, respectively. E6 is a ubiquitin ligase, specifically an E6-AP ubiquitin protein ligase, which attaches to and directs the 26S proteasome to degrade the tumor suppressor protein p53. This inactivation of p53 results in the loss of its function as a cell cycle checkpoint, enabling genetic mutations to accumulate and increasing cell growth and division (52). E7 is an inhibitor of cyclin-dependent kinase (CDK). It binds to and inactivates the tumor suppressor protein retinoblastoma protein (Rb), liberating the transcription factor E2F from its inhibitory binding to Rb and allowing for an increase in the expression of genes involved in cell cycle progression (53). Consequently, this leads to the lack of apoptotic induction and uncontrolled cell cycle progression.

The connection between p53 and MDM2 is essential for controlling cell growth and apoptosis but with different processes in HPV^+/-^ related cancers. Although mutant p53 follows the same routes as wild-type p53, in HPV^-^ HNSCC the p53 mutation prevents transcription activation from occurring downstream and affects the p53 target genes. However, in HPV^+^ HNSCC the process of HPV oncoprotein-mediated disruption of the cell cycle is different. *In vivo* investigations have demonstrated that MDM2 and HPV E6 utilize distinct methods to degrade p53 (42). HPV E6 binds p53 for degradation when the usual MDM2-p53 ubiquitination pathway is dormant (e.g: post-DNA damage). In contrast, amplification or overexpression of MDM2 might inhibit the p53 pathway by directly binding and masking p53′s transactivation domain or by serving as an E3 ubiquitin ligase for p53 destruction (39, 40). MDM2 interacts with HPV16’s E2 to activate the HPV16 promoter mechanically, suggesting that HPV16’s E2 may actively recruit MDM2 to the HPV promoter for cooperative involvement in HPV’s E2-activated gene production (43). In another hand, HPV E6 oncoprotein operates by activating the ubiquitin-dependent proteolysis pathway to degrade p53, leading to the loss of G2/M checkpoint control. Indeed, E6-associated protein (E6-AP) is essential to enable the breakdown of E6 (44), and thus it functions as an E3 ubiquitin ligase (45). Consequently, the HPV E6 protein binds to the resulting trimeric complex between p53, E6, and E6-AP which arises in the ubiquitination and eventual degradation of p53 by the proteasomes. It is worth noting that the simultaneous suppression of E6-AP and MDM2 expression did not affect the p53 accumulation. When E6-AP expression is suppressed, MDM2 might not be the primary regulator of p53 degradation in the HPV^+^ cells. Indeed, the MDM2 negative feedback loop remains inert in HPV^+^ cells due to the presence of E7. HPV E7 oncoprotein binds to pRb, resulting in nuclear translocation of E2F and stimulation of S-phase transition. In addition, Rb downregulation leads to a lack of feedback inhibition and an increase in p16^INK4A^ expression (46, 47, 2), which may then stimulate the ARF tumor suppressor gene activation (48, 49). It has been reported that ARF prevents the degradation of p53 in HeLa cells by binding to MDM2 and accelerating MDM2 turnover (50). Moreover, more evidence suggested that ARF decreases MDM2’s ubiquitin ligase function (51). In light of this, the MDM2 feedback loop is assumed to be inhibited in the presence of E7 expression. Both E6 and E7 play crucial roles in the development of HPV-associated malignancies by deactivating essential tumor suppressor proteins, promoting genetic mutation accumulation and uncontrolled cell proliferation. We summarized the p53 signaling pathway in cells with HPV^-^ cells (mutant p53 cells) and HPV^+^ cells in **Figure 8**.

In contrast to HPV^-^ HNSCC, HPV^+^ HNSCC has a good prognosis, with 5-year survival rates averaging between 75% and 80%, since these cancers react better to chemotherapy and radiation (54). Vlatković et al. showed that using the Cox multivariate analysis of overall survival according to clinical parameters and individual gene expression revealed MDM2 as a significant predictor (p <0.03; OR = 0.63; 95% CI = 0.41–0.96) of overall survival (55). Their findings were consistent with our study showing that a high level of MDM2 expression (*p* = 0.002) is correlated with improved overall survival in HPV^+^ HNSCC patients compared to HPV^-^ HNSCC patients (*p* = 0.263). The inverse relationship between HPV status and high *MDM2* expression is consistent with several studies showing that HPV^+^ HNSCC patients appear to have a significantly improved response to radiotherapy and chemotherapy, in addition to a lower risk of second primary cancers than the HPV^-^ (56). Although the particular mechanism is complicated and not fully understood, several hypotheses could explain this inverse association including the fact that the genome of HPV^+^ cancer cells seems to be slighter unstable, in addition, that HPV^+^ cells suffering from hypoxia can be more easily activated to apoptosis, or alternatively, the treatment may improve the local immunity, which may then favour the HPV elimination and tumour regression (57–59). These hypotheses provide considerable insight into the prognostic role of *MDM2* in HPV^+^ HNSCC patients, however, further studies are needed to accurately understand the association between HPV status, MDM2 expression and patients’ outcomes.

We also evaluated the correlations between MDM2 protein expression and different demographic clinical characteristics of the HNSCC patients, using Pearson’s correlation (p ≤ 0.05), to show a positive association between MDM2 expression and HPV status (*Pearson correlation* = 0.28). Additionally, we revealed a negative correlation between MDM2 expression and smoking (*Pearson correlation* = -0.12) in HPV-associated OSCC patients. Notably, the effects of MDM2 genetic alterations were more prominent in never-smokers and never-drinkers (60, 2). Chen et al. further showed that the modifying impact of MDM2 mutations on the incidence of OSCC is linked to HPV16 L1 seropositivity, which was greater in never-smokers than in ever-smokers and in never-drinkers than in ever-drinkers (61). L1 is the principal structural protein of the HPV virus and plays a crucial function in the assembly of viral particles. The interaction between mutant MDM2 and the L1 protein of HPV16 results in an increase in the L1 protein’s stability and the assembly of virus-like particles, which can augment the virus’ infectiousness. In addition, the interaction between mutant MDM2 and L1 protein can increase the activity of the E6 oncoprotein and the degradation of p53, both of which can contribute to cancer development and progression (61). Consequently, we propose that when evaluating the modifiable effects of MDM2 mutations on the risk associated with HPV16 L1 seropositivity, smoking and alcohol consumption may also need to be considered.

Multiple pieces of research have focused on the relationship between MDM2 and p53 as a promising cancer treatment target. When MDM2-binding compounds were introduced, disruption of this connection in several cancer cell lines led to apoptosis-mediated cell death. Cancer cell cycle arrest and apoptosis are induced by disrupting the MDM2-p53 connection, which leads to an increase in p53 (24, 62). Importantly, targeting overexpressed MDM2 in cancer cell membranes causes the breakdown of cell membrane integrity, resulting in necrosis of cancer cell membranes. Significantly, along with the expanding use of genomic profiling for customized cancer treatment, MDM2 continues to function as a potentially effective molecular therapeutic target (63, 64). This molecular-based method may in part drive the future of cancer therapy to customize tailored treatment options, therefore, strengthening our arsenal to enhance patient outcomes in HPV-associated HNSCC.

Moreover, we showed that p53-target genes such as *CDKN2A, NOTCH1* and *PCNA*, were up-regulated in HPV^+^ HNSCC patients, however, *CAV1, CD44*, and *RTN4* were up-regulated in HPV^-^ HNSCC, suggesting that changes in these markers are expected to disturb the cell cycle and facilitate carcinogenesis either directly or indirectly in the HPV-associated HNSCC. Given that in the case of HPV^+^ HNSCC, the p53 is inactivated by E6-E6AP, thus, we can suggest that the overexpression of the identified p53 target genes could be explained by the involvement of other pathways outside of p53, including the activation of alternative signaling pathways, such as the PI3K/Akt pathway (65). In addition, other transcription factors, including as c-Myc and Sp1, can bind to and activate the promoters of p53 target genes in the absence of functioning p53 (66). While others studies provide additional support that miRNA-mediated downregulation of E6-associated proteins (such as E6-AP) may result in p53 pathway reactivation (67).

Furthermore, the multiplicity of the human cancer cells reveals the inactivation of the P53 pathway. P53 controls several target genes that play key roles in the arrest of the cell cycle, DNA repair, cellular senescence, and apoptosis. Therefore, P53-reactivation approaches by targeting the key p53-target genes can also play a critical role in oral cancer therapy. Currently, as an imperative step forward in cancer therapy, different molecule drugs targeting MDM2-p53 which are in the clinical trials stage could restore p53 antitumor activity by preventing the MDM2-P53 interaction in solid and hematologic tumors (68). From a future perspective, additional validation of the identified biomarkers including *EZH2, PCNA, STAT5A* and *TYMS*, that could be considered as p53-downstream genes will help in the diagnosis and prognosis of HPV^+^ HNSCC. Further studies, which take the sub-classification of HPV^+^ cases into account, will need to be undertaken, and thus further molecular validation is warranted to assess the HPV genotype and the particular mutations on p53, which can help correlate the mutations with the expression profiles of the identified p53 target genes.

In conclusion, our study reported a comprehensive bioinformatics analysis of DEGs between HPV^+/-^ HNSCC, which identified several p53 target genes that may have the potential to serve as reliable molecular biomarkers for the diagnosis and/or prognosis of HPV-associated HNSCC. Importantly, *MDM2* was further validated to be up-regulated in HPV^+^ compared to HPV^-^ HNSCC, and its high expression was related to increased overall survival. *MDM2* may act as a prognostic biomarker and potential therapeutic target in HPV-associated HNSCC. Further studies are deserved to explore the biological functions of these p53 target genes to elucidate the underlying molecular mechanisms in the pathogenesis of HPV-associated HNSCC.

## Data availability statement

The datasets presented in this study can be found in online repositories. The names of the repository/repositories and accession number(s) can be found below: GEO: GSE3292, GSE6791 and GSE55542, TCGA and GTex: GTEx Analysis V8 (dbGaP Accession phs000424.v8.p2).

## Ethics statement

The studies involving human participants were reviewed and approved by the Research Ethics Committee at Tawam Hospital (REC: AA/AJ/556). Written informed consent for participation was not required for this study in accordance with the national legislation and the institutional requirements.

## Author contributions

Conceptualization: AB, RH and NA-R. Data curation: AB and NA-R. Bioinformatics analysis: AB. Funding acquisition: RH and NA-R. Methodology: AB, DF, MA, AQ, and ZA. Supervision: RH and NA-R. Writing - original draft: AB and NA-R. Writing, review and editing: AB, ZA, RH and NA-R. All authors contributed to the article and approved the submitted version.
