# Supplementary material for: Identification of p53-target genes in human papillomavirus-associated head and neck cancer by integrative bioinformatics analysis
Source: Front Oncol. 2023 Apr 4;13:1128753. doi: 10.3389/fonc.2023.1128753 (PMC10110890; doi:10.3389/fonc.2023.1128753)
Supplement: Supplementary file 1 [file Presentation_1.pdf]

Supplementary Figures:

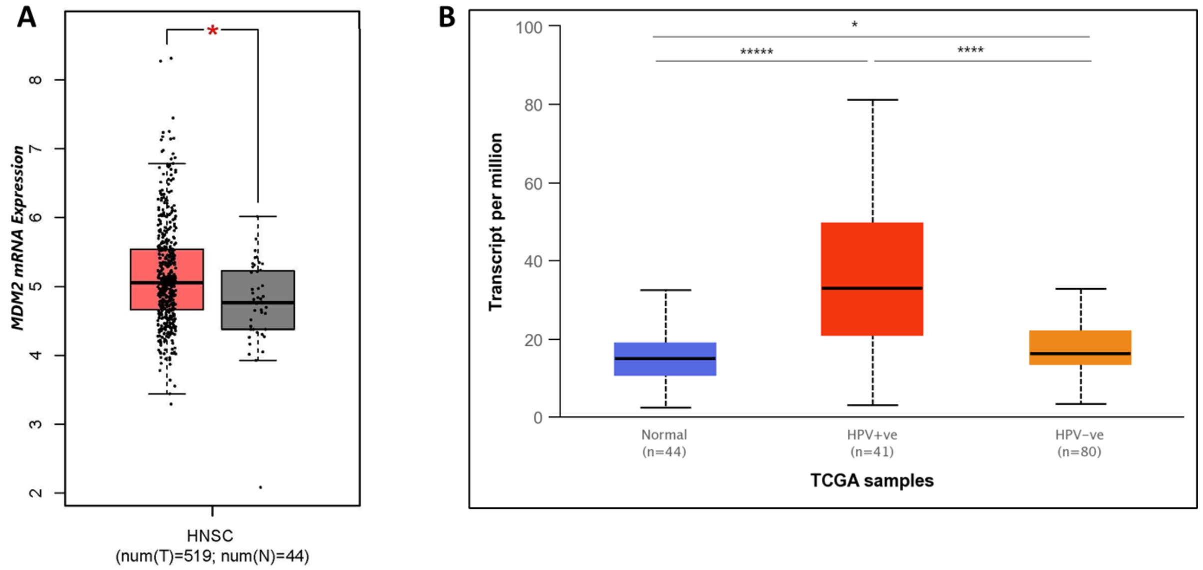

Supplementary Figure 1: Validation of *MDM2* mRNA expression in a large cohort of HNSC patients. A) Box plot shows the mRNA levels of *MDM2* expression in Head and Neck squamous cell carcinoma (HNSC) tissues and normal tissues, using TCGA and GTEx databases in GEPIA. B) Expression of *MDM2* in HNSC based on the HPV status compared to normal. $*p < 0.05$, $****p <$ 1E-05, $*****p <$ 1E-07

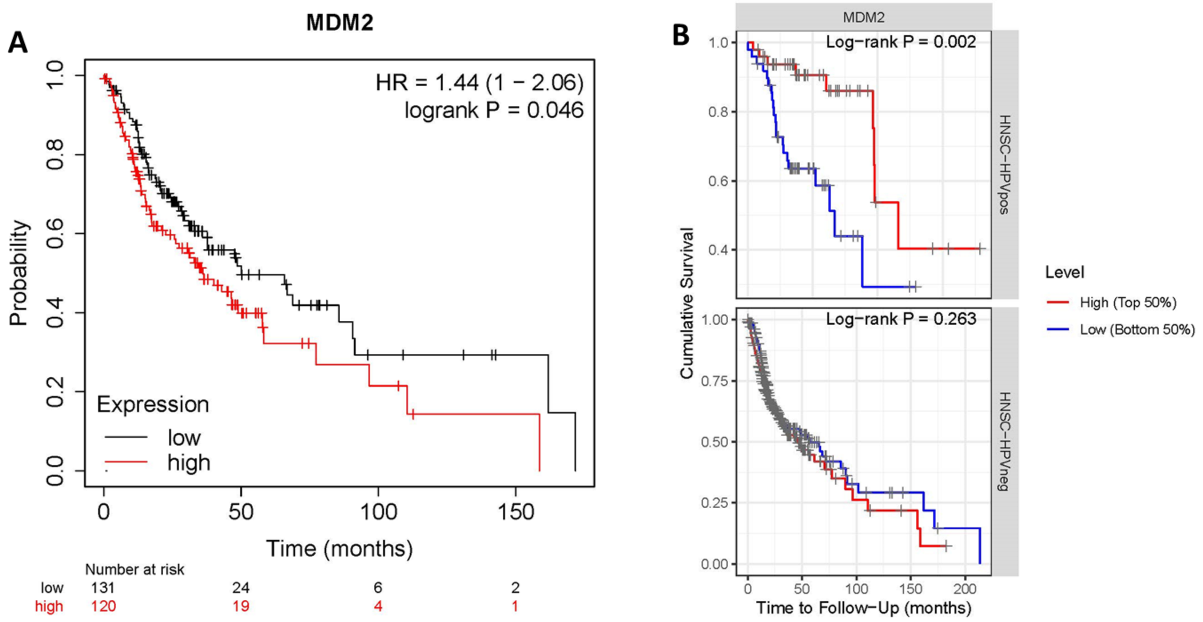

Supplementary Figure 2: Overall survival of MDM2 in HNSC patients using Kaplan-Meier plotter. A) The relatively high expression of MDM2 is correlated with a poor prognosis of HNSC. The survival analysis was calculated among 500 HNSC patients. B) The top plot shows that the relatively high expression of MDM2 is significantly correlated with increased prognosis of HPV+ HNSC patients, however, the bottom plot displays no significant influence on HNSC HPV- patients' overall survival. The expression of *MDM2* was calculated

among 98 HPV-positive HNSC patients and 420 HPV-negative HNSC patients. Data are presented as the hazard ratio with a 95% confidence interval. The red plots show high expression while the black/blue plots show low expression of MDM2 in HNSCC patients.
