# Supplementary material for: Identification of p53-target genes in human papillomavirus-associated head and neck cancer by integrative bioinformatics analysis
Source: Front Oncol. 2023 Apr 4;13:1128753. doi: 10.3389/fonc.2023.1128753 (PMC10110890; doi:10.3389/fonc.2023.1128753)
Supplement: Supplementary file 2 [file Table_1.pdf]

Supplementary Tables

Supplementary Table 1: The list of datasets used in the study

#	Dataset ID	Cancer Type	HPV^{+} samples	HPV^{-} samples	Population
**1**	**GSE3292**	Head and Neck Squamous Cell Carcinoma	8	28	American
**2**	**GSE6791**	Head and Neck Cancers	16	26	American
**3**	**GSE55542**	Oropharyngeal Squamous Cell Carcinoma	20	16	European American and African American patients.
	**Total**	114	44	70	

Supplementary Table 3: List of antibodies used in this study

#	Antibody	Dilution	Clone	Vendor	Catalog number	Platform	Protocol
**1**	MDM2	1:100	2A-10	Abcam	Ab16895	Benchmark-Ultra	PT-Link-pH6/FLEX/20min.
**2**	P16	RTU	E6-H4	Ventana	705-4793	Benchmark-Ultra	CC1-36/UV-DAB/16min.
**3**	P53	RTU	DO-7	Ventana	790-2912	Benchmark-Ultra	CC1-64/UV-DAB/20min.

Supplementary Table 6: The Top 20 Hub genes in the PPI network ranked by the Degree method

Rank	Ensembl ID	Name	Description	Score
**1**	ENSP00000368458	PCNA	proliferating cell nuclear antigen	24
**2**	ENSP00000216122	MCM5	minichromosome maintenance complex component 5	19
**3**	ENSP00000480987	MCM3	minichromosome maintenance complex component 3	18
**3**	ENSP00000265056	MCM2	minichromosome maintenance complex component 2	18
**5**	ENSP00000368349	POLA1	DNA polymerase alpha 1, catalytic subunit	17
**6**	ENSP00000363017	RPA2	replication protein A2	16
**7**	ENSP00000264156	MCM6	minichromosome maintenance complex component 6	13
**7**	ENSP00000350491	PRIM1	DNA primase subunit 1	13
**7**	ENSP00000408295	RFC5	replication factor C subunit 5	13
**10**	ENSP00000362592	RBBP4	RB binding protein 4, chromatin remodeling factor	12
**10**	ENSP00000370376	DUT	deoxyuridine triphosphatase	12

**12**	ENSP00000315644	TYMS	thymidylate synthetase	11
**13**	ENSP00000300738	RRM1	ribonucleotide reductase catalytic subunit M1	10
**14**	ENSP00000320147	EZH2	enhancer of zeste 2 polycomb repressive complex 2 subunit	9
**14**	ENSP00000263274	LIG1	DNA ligase 1	9
**14**	ENSP00000301280	CHAF1A	chromatin assembly factor 1 subunit A	9
**17**	ENSP00000369424	RBBP7	RB binding protein 7, chromatin remodeling factor	8
**17**	ENSP00000258149	MDM2	MDM2 proto-oncogene	8
**19**	ENSP00000316578	SUZ12	SUZ12 polycomb repressive complex 2 subunit	7
**19**	ENSP00000286398	SMC2	structural maintenance of chromosomes 2	7
